# Supplementary material for: Synthesis, X-ray Characterization and Density Functional Theory (DFT) Studies of Two Polymorphs of the α,α,α,α, Isomer of Tetra-p-Iodophenyl Tetramethyl Calix[4]pyrrole: On the Importance of Halogen Bonds
Source: Molecules. 2020 Jan 10;25(2):285. doi: 10.3390/molecules25020285 (PMC7024273; doi:10.3390/molecules25020285)
Supplement: Supplementary file 1 [file molecules-25-00285-s001.pdf]

molecules

SUPPORTING INFORMATION

Synthesis, X-Ray Characterization and Density Functional Theory (DFT) Studies of Two Polymorphs of the $\alpha,\alpha,\alpha,\alpha$, Isomer of Tetra-*p*-Iodophenyl Tetramethyl Calix[4]pyrrole: On the Importance of Halogen Bonds

Dragoș Dăbuleanu [1,2], Antonio Bauzá [3], Joaquín Ortega-Castro [3], Eduardo C. Escudero-Adán [1], Pablo Ballester [1,4*] and Antonio Frontera [3,*]

1 Institute of Chemical Research of Catalonia (ICIQ), The Barcelona Institute of Science and Technology (BIST), Avinguda Països Catalans, 16, 43007 Tarragona, SPAIN; ddabuleanu@iciq.es(D.D.); eescudero@iciq.es(E.C.E.-A.)

2 Departament de Química Analítica i Química Orgànica, Universitat Rovira i Virgili, carrer Marcel•li Domingo, 1, 43007 Tarragona, SPAIN

3 Departament de Química, Universitat de les Illes Balears, Crta. de Valldemossa km 7.5, 07122 Palma de Mallorca (Baleares), SPAIN; antonio.bauza@uib.es(A.B.); joaquin.castro@uib.es(G.O.-C.)

4 Catalan Institution of Research and Advanced Studies (ICREA), Passeig Lluís Companys, 23, 08018 Barcelona, SPAIN

* Correspondence: pballester@iciq.es (P.B.); toni.frontera@uib.es (A.F.)

Synthesis of tetraiodo calix[4]pyrrole:

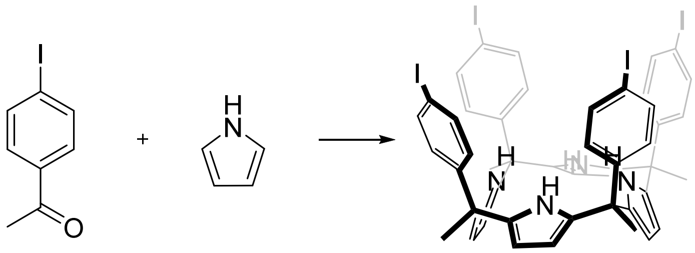

The reaction conditions are an optimized version of previously reported protocol.[1] In a 1000-mL round bottom flask, 500 mL of a dichloromethane solution of 4′-iodoacetophenone (10 g, 40.6 mmol, 0.08M) were added. Next, 3.5 mL of HCl (aq. 36%) (40.6 mmol) were added dropwise to the above solution. Using an automatic injector pump syringed, a solution of 100 mL of DCM containing 2.82 ml of pyrrole (40.6 mmol) was added to the above reaction mixture over the course of 24 hours. The reaction flask was protected from light with aluminum foil and the reaction mixture was left stirring for a further 48 hours at room temperature. A precipitate formed during the reaction time, which was composed of polymeric/oligomeric byproducts. This solid was filtered and washed with 300 mL of methanol to ensure that only the polymeric products remain on the filter. The methanol solution was combined with the DCM crude obtained from the reaction and concentrated under reduced pressure to afford a brown solid. The $\alpha,\alpha,\alpha,\alpha$ isomer was obtained as a brownish solid (4.55 g, 36%) after silica column chromatography purification of the reaction crude using a 40:60 mixture of DCM:hexane as mobile phase.

^{1}H NMR (500 MHz, $CDCl_3$) δ (ppm): 7.56 (d, *J*=8.5Hz, 8H), 7.53 (br, 4H), 6.84 (d, *J*=8.5Hz, 8H), 5.72 (d, *J*=2.7Hz, 8H), 1.93 (s, 12H).**^{13}C NMR** (125 MHz, $CDCl_3$) δ (ppm):147.9, 136.8, 135.9, 129.6, 106.7, 92.2, 44.6, 27.9. **IR (film)** (cm^{-1}): 3427, 3414, 2975, 2251, 1581, 1482, 1426, 1393, 1367, 1267, 1220, 1187, 1100, 1082, 10, 1033, 1006, 829, 819, 762, 721, 710, 657, 577, 532, 498.

1. [1] Galan, A.; Aragay, G.; Ballester, P. A chiral "Siamese-Twin" calix[4]pyrrole tetramer. *Chem. Sci.* **2016**, *7*, 5976-5982.

Characterization of tetraiodo calix[4]pyrrole:

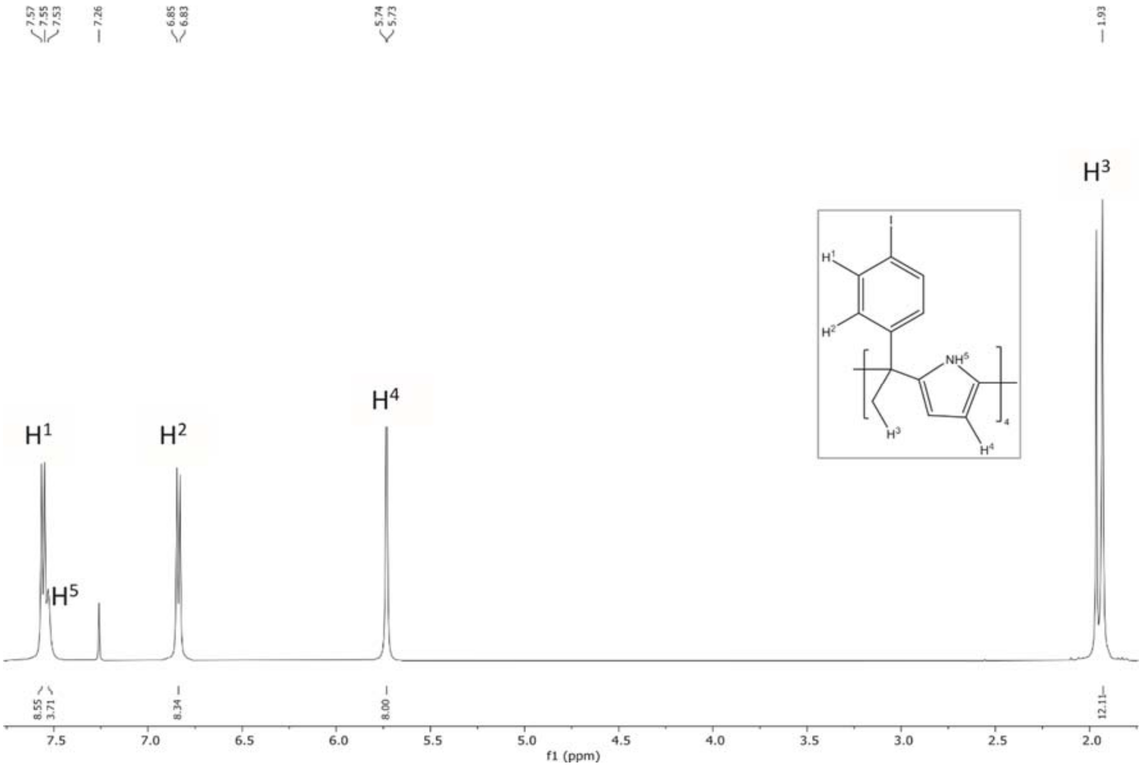

Figure 1. Selected region of ^{1}H NMR spectrum with the assignment of the proton signals of the calix[4]pyrrole.

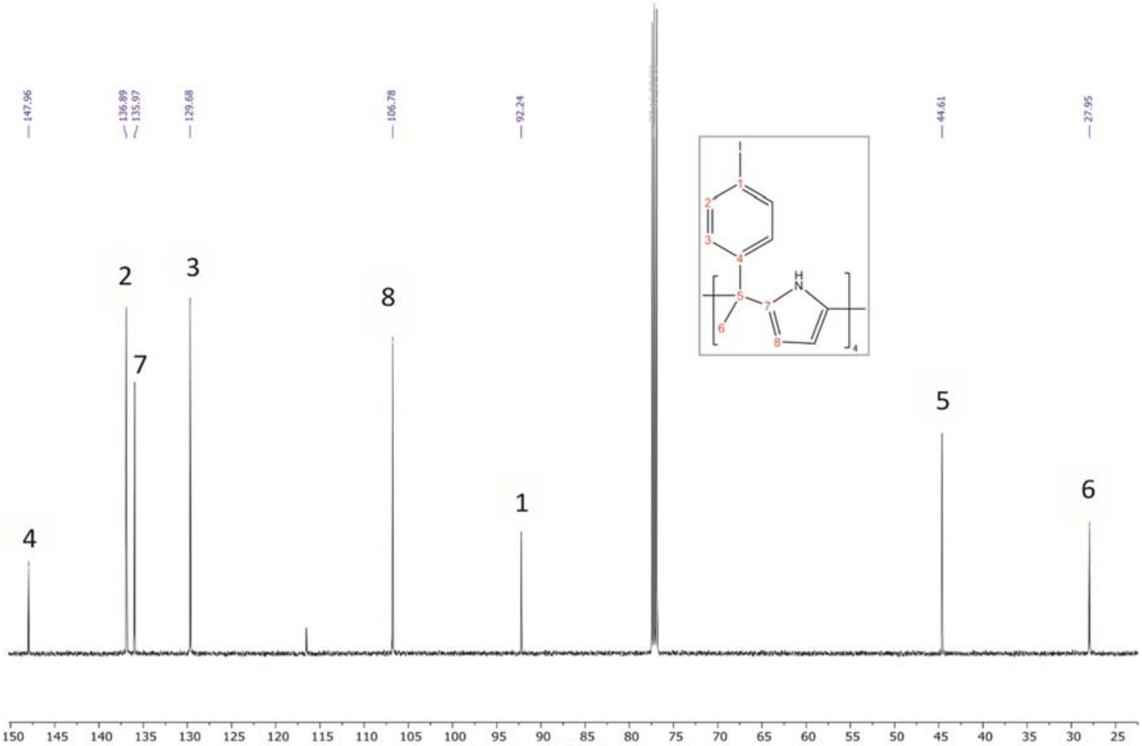

Figure 2. Selected region of ^{13}C NMR spectrum with the assignment of the signals to the calix[4]pyrrole structure.

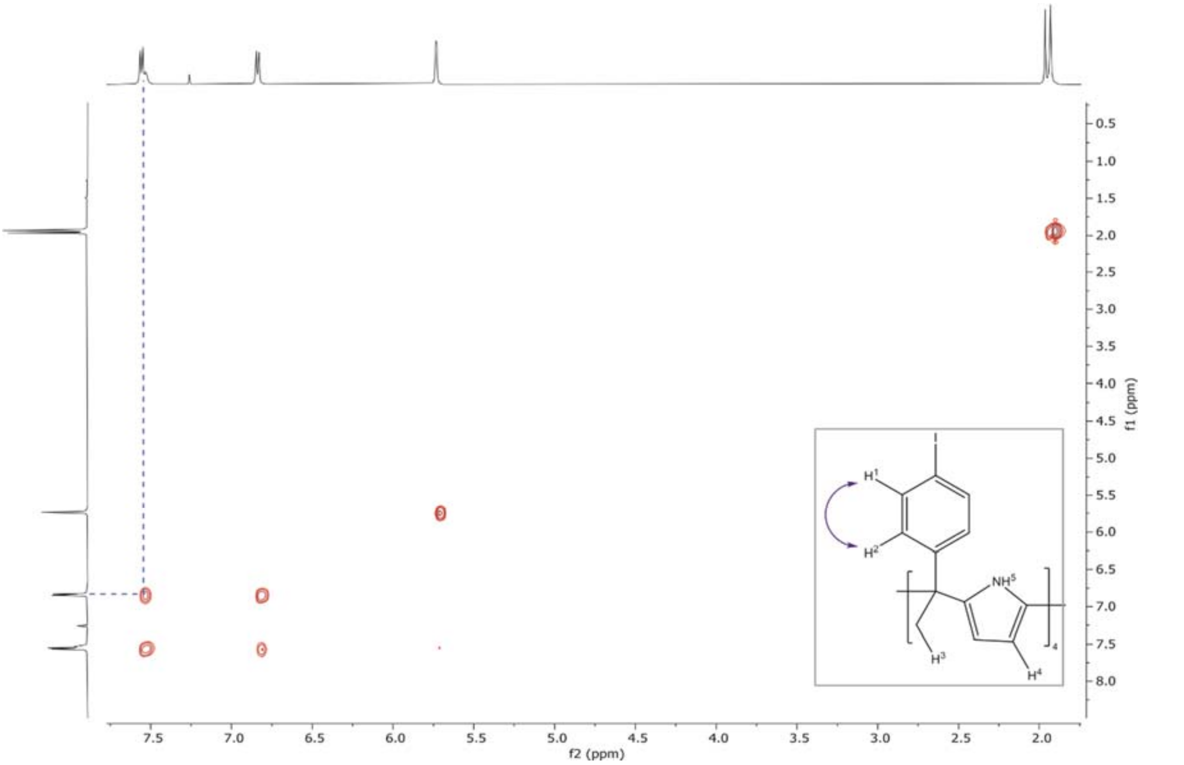

Figure 3. Selected region of COSY NMR experiment (COSY45TBFO) in $CDCl_3$. Inset contains the assignment of the correlations found (e.g., H^1-H^2).

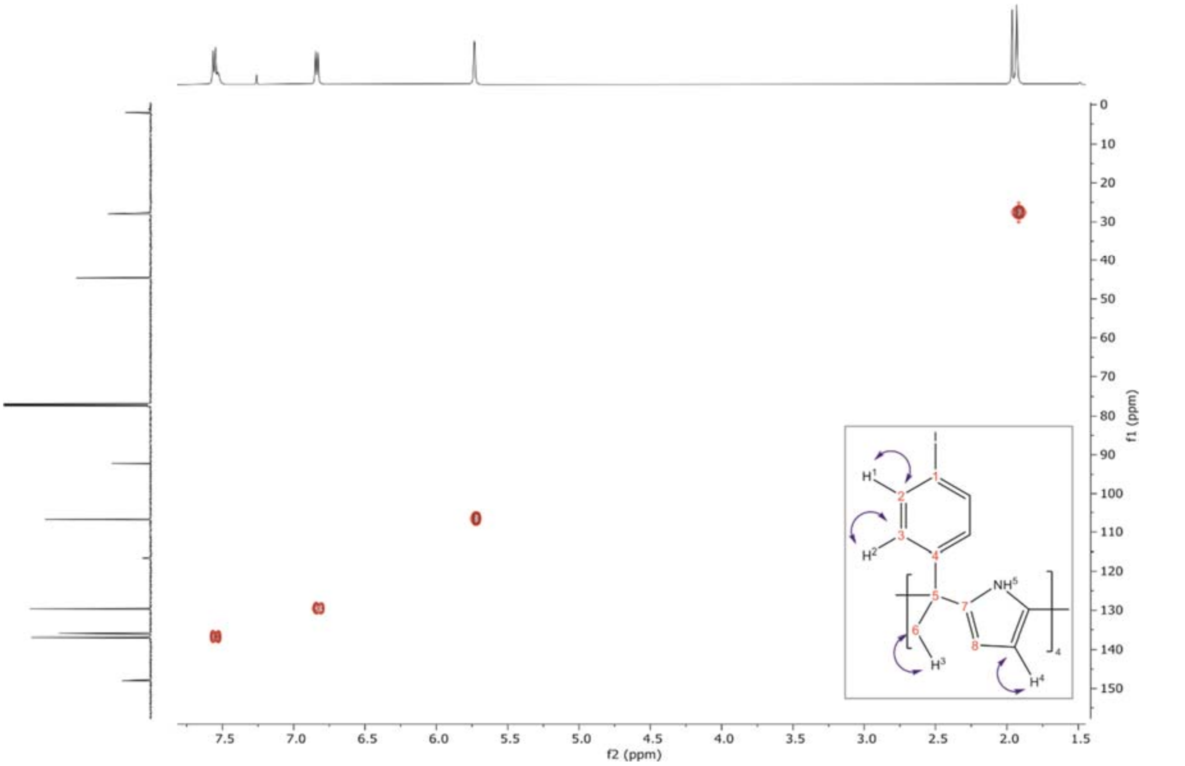

Figure 4. Selected region of HMBC NMR experiment (HMBCTBFO) in $CDCl_3$. Inset contains the assignment of the correlations found (e.g. H^1-C2, H^2-C3, H^3-C6, H^4-C8).

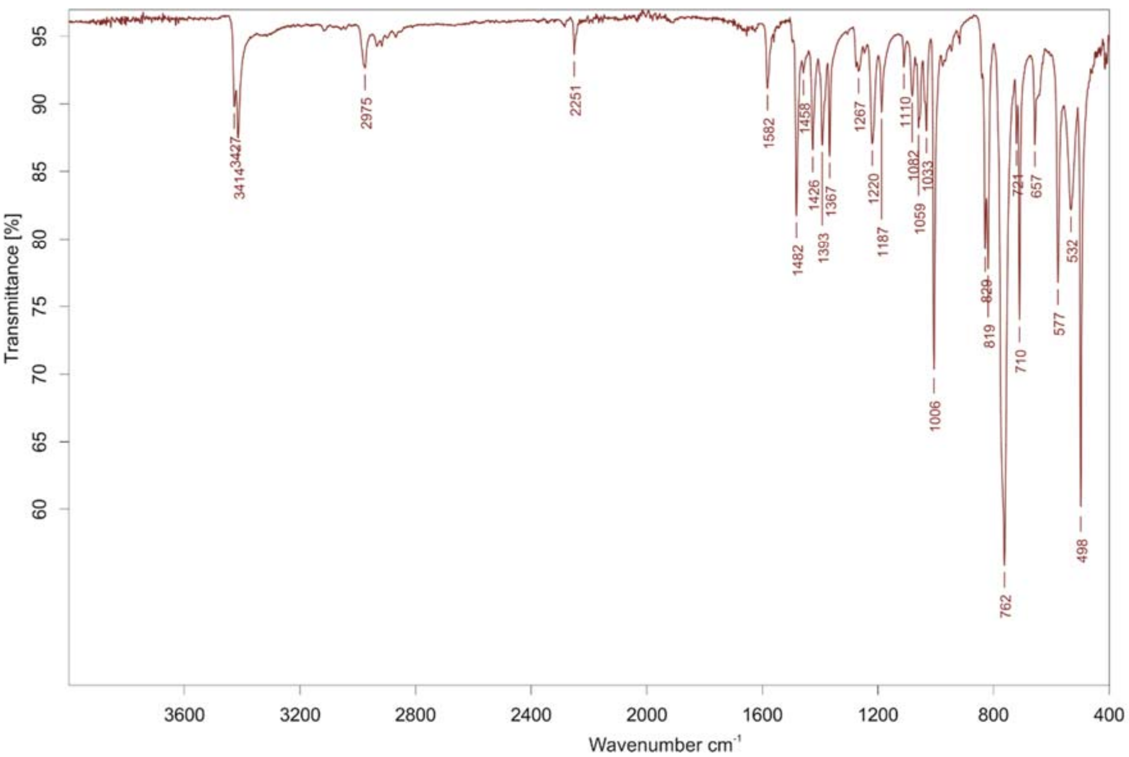

Figure 5. IR (film) spectrum of the calix[4]pyrrole.
